# Editorial: Novel Insights Into the Multifaceted Mitotic Kinases

**DOI:** 10.3389/fcell.2019.00051

**Published:** 2019-04-09

**Authors:** Sabine Elowe

**Affiliations:** ^1^Department of Pediatrics, Faculty of Medicine, Axe of Reproduction, Mother and Infant Health, Université Laval, Quebec City, QC, Canada; ^2^Research Center of the CHU de Québec, Quebec City, QC, Canada

**Keywords:** mitosis, meiosis, SAC, kinases, phosphatase

Mitosis, the shortest stage of the cell cycle, is arguably the most dramatic and visually beautiful stage in the life of a cell. In higher eukaryotes, it requires complete cellular restructuring, including the disassembly of many organelles and subcellular structures such the nucleus, the endoplasmic reticulum and the cilia to name but a few. Conversely, other structures which are highly specialized for this phase of the cycle, in particular the mitotic spindle and kinetochores -proteinaceous complexes that form the microtubule interaction interface- are only assembled during mitosis. It is now well recognized that these dramatic structural changes are orchestrated through a coordinated series of post-translational modifications, the best characterized of which are serine and threonine phosphorylations, driven by kinase activity. Indeed, more than three-quarters of all proteins in a cell are phosphorylated at one or more sites in human cells, with the highest occupancy of phosphorylation sites seen in mitosis (Olsen et al., 2010; Sharma et al., 2014).

Mitotic kinases is the name given to the group of kinases that control entry into and the progression through the mitotic stage of the cell cycle. This group broadly includes the master mitotic kinase cyclin-dependent kinase 1 (CDK1), the polo-like kinase 1(PLK1), the Greatwall kinase (Gwl), the atypical kinase Haspin, the Aurora Kinases (AurA, AurB, and the germ cell-specific AurC), the NIMA-related kinases (NEKs), and the spindle checkpoint kinases Monopolar Spindle 1 (Mps1) and Budding Uninhibited by Benzimidazole 1 (BUB1). Not surprisingly, and given the success of many kinase inhibitors in the clinic, the mitotic kinases have attracted much attention as cancer-specific therapeutic targets (Dominguez-Brauer et al., 2015). However, to date, the clinical outcomes have been disappointing and one could argue that this is due at least in part to a lack in our understanding of their regulation and their functions during mitosis and beyond. In this frontiers research topic on *the recent insights into these multifaceted mitotic kinases*, we compile contributions from up-and-coming researchers in the field and underline novel observations related to the mitotic kinase function and regulation that are emergent in the recent literature.

The kinase fold is one of the best studied structural folds and yet it is the unique features of kinases that determine their regulation and activation in space and time. Mitotic kinases however are largely atypical and many do not belong to any of the major kinase families. As such, it is often difficult to predict their mechanisms of regulation. The minireview by Welburn and Jeyaprakash, summarizes our current knowledge of the common and unique structural features of a number of mitotic kinases and discuss specific mechanisms of activation (such as allostery and phosphorylation), and mechanisms of substrate selectivity (including subcellular localization, consensus target sequences and substrate priming).

Some of the most poorly understood mitotic kinases are the NEKs which were originally identified in the 1980s in the fungus *Aspergillus nidulans*, and which include 11 members in humans (Osmani et al., 1988; Fry et al., 2012). The NEKs are a pleitropic family of kinases with several roles throughout the cell cycle. In mitosis however, they regulate different events including centrosome separation and spindle assembly upon mitotic entry as well as cytokinesis. In the comprehensive review by Fry et al., the authors describe how NEK kinases achieve these functions through the targeting of proteins that regulate the microtubule cytoskeleton and shed novel insights into mechanisms of regulation of their enzymatic activity.

Unlike the NEKs, AurB, the catalytic arm of the chromosomal passenger complex (CPC) has been a subject of intense study in recent years and many substrates have been identified that communicate the CPC's role in correction of attachment errors between spindle microtubules and kinetochores. Although its function is widely thought to be dependent on its dynamic localization (from the inner centromere in early mitosis, through to the central spindle and cleavage furrow in cytokinesis), recent work has questioned the importance of this characteristic localization pattern to the efficacy of CPC and in particular AurB function (Campbell and Desai, 2013; Hengeveld et al., 2017). In their review Hindriksen et al. provide a detailed discussion of the mechanisms regulating AurB and CPC localization, and in particular the role that two other mitotic kinases Haspin and BUB1 play in this process, and debate the significance of CPC targeting to the centromere.

Meiosis is a specialized from of cell division, where haploid cells are generated from dipoloid cells after two reductionist cell divisions. Although much of the machinery is shared between mitosis and meiosis, additional roles are undertaken by kinase signaling pathways during meiosis to ensure proper chromosome segregation under these specific conditions. Marston and Wassmann elegantly compare kinase function in somatic and germ cells, focusing on meiosis in budding yeast and in mouse oocytes. They highlight both similarities and differences between these systems, and describe how the additional meiotic functions of kinases help maintain the correct ploidy in gametes where chromosome segregation is erroneous.

Although the core cohort of mitotic kinases is now clear, there are likely additional supporting kinases that contribute to cell division but that are nonessential under normal growth conditions. In the original article by Rusin et al., the authors use quantitative proteomics techniques to identify novel mitotic functions and substrates for one such kinase, the ubiquitously expressed Casein Kinase 2 (CK2). CK2 is active throughout the cell cycle, and as such is not classically considered a mitotic kinase, although it is known to be required for progression into mitosis in budding yeast and may regulate chromosome condensation at mitotic entry and mitotic progression through spindle substrates (Glover, 1998; Takemoto et al., 2006; St-Denis et al., 2009, 2011). The authors now confirm the role of CK2 in chromosome condensation and chromatin organization, and reveal how the activity of CK2 is counteracted by the Serine/Threonine phosphatase PP6 (Rusin et al.; Rusin et al., 2015).

Finally, what goes up, must come down. Indeed, it is difficult to discuss mitotic kinase signaling without implicating mitotic dephosphorylation which, perhaps not surprisingly, has attracted considerable recognition very recently as a major mechanism for mitotic exit. Two thought-provoking and exciting reviews from Saurin and Nasa and Kettenbach explore the functions of phosphatases in mitosis. Nasa and Kettenbach detail the literature regarding the role of phosphoprotein phosphatases from mitotic entry to exit with emphasis on their regulation of and by mitotic kinases. The authors also make the intriguing argument that despite early misconceptions, emerging data argue for a certain level of selectivity and specificity of these phosphatases for their targets. This idea is supported by recent publications demonstrating distinct localization patterns of individual B56 subunits during prometaphase (Nijenhuis et al., 2014; Vallardi et al., 2019). Finally, in a thought-provoking piece, Saurin discusses in great detail how (and why) kinase and phosphatase networks are intimately intertwined during mitosis and the implications of this cross-talk. He discusses strategies on how to experimentally address kinase-phosphatase signaling specificity at the outer kinetochore and to distinguish direct from indirect signaling. The emergent picture illustrates the exquisite sensitivity of kinetochore function to the counterbalancing activities of these enzymes.

While many of the initial discoveries in the mitotic signaling field have set the pace and formed the global picture of how signaling networks are integrated in early mitosis, many of the finer details of how the mitotic kinases are themselves regulated and how they regulate downstream signaling are just now starting to emerge. As the saying goes, the devil is in the details.

## Author Contributions

SE and Anna Santamaria Margalef would like to thank all contributing authors and reviewers for their support to the Research Topic.

### Conflict of Interest Statement

The author declares that the research was conducted in the absence of any commercial or financial relationships that could be construed as a potential conflict of interest.
